# The Effects of a Moderate Exercise Program on Knee Osteoarthritis in Male Wistar Rats

**Published:** 2013-05

**Authors:** Mohammad Fallah Mohammadi, Akbar Hajizadeh Moghaddam, Hosein Mirkarimpur

**Affiliations:** 1 Department of Sport Pathology and Corrective Exercises, School of Physical Education and Sport Sciences, University of Guilan, Rasht, Iran; 2 Department of Biology, Faculty of Sciences, University of Mazandaran, Babolsar, Iran; 3 Department of Sport Pathology and Corrective Exercises, School of Physical Education and Sport Sciences, University of Tehran, Tehran, Iran

**Keywords:** Exercise, Monosodium Iodoacetate, Rat’s Knee Osteoarthritis

## Abstract

***Objective(s):*** Osteoarthritis (OA) or degenerative joint disease is the commonest form of arthritis and can lead to joint pain, decrease in joint’s range of motion, loss of function, and ultimately disability. Exercise is considered as one of the non-pharmacological treatments of OA. But the effects of exercise on knee joint cartilage remain ambiguous. The aim of the present study was to investigate the effect of a four-week moderate treadmill exercise on rats’ knee osteoarthritis.

***Materials and Methods:*** Eighteen male Wistar rats (173 ± 1 g, 8 weeks old) were randomly divided into three groups (n = 6): Intact control, monosodium iodoacetate (MIA) only (OA), and training. The osteoarthritis model was induced by intra-articular injection of monosodium iodoacetate (MIA). Subjects followed a moderate-intensity exercise program for 28 days. Rats were killed after 28 days and histological assessment was done on their knee joints. One-way ANOVA (*P*<0.05) and post-hoc Tukey test was used for the statistical analysis.

***Results:*** Histological assessment on 3 measurements of, depth ratio of lesions (*P*=0.001), total cartilage degeneration width (*P*=0.001), and significant cartilage degeneration width (*P*=0.001), demonstrated that moderate exercise for 4 weeks could surprisingly almost treat OA symptoms of rats’ knee joints.

***Conclusion:*** The findings of the present study indicate that a moderate treadmill exercise program exert a beneficial influence on rats’ knee osteoarthritis.

## Introduction

Osteoarthritis (OA) is considered to be the commonest form of arthritis and can result in joint pain, decrease in joint range of motion, loss of function and disability (1). It has been reported that 45 to 75% of people older than 55 years have early symptoms of OA and this is the reason why OA is regarded as a global concern (2). Among elderly, knee OA is one of five major causes of disability in developed countries and it is estimated that nearly 100000 people in the U.S. are unable to walk due to knee or hip OA (3). From 1995 to 2005, Patients diagnosed with OA have increased by 6 million and it is expected that the number of people disabled due to OA will be doubled by the year 2020 (4, 5).

Several risk factors related to OA have been studied, among which, age, joint injury, nutrition, occupation, joint deformation, genetic factors and intense exercise are more common (6,7). There have been several studies in the field of sports medicine that consider knee OA as the most important disease that can occur after injury to this joint (8-11). This emphasizes the importance of addressing long-term consequences of sports injuries.

Although its nature and symptoms are well defined and its related risk factors have been studied precisely, there is no known cure for OA (12, 13). The treatment modalities for OA include non-pharmacological and pharmacological treatments and ultimately surgery. Non-pharmacological treatments involve physiotherapy, aerobic and strength training exercises, weight loss, wearing braces and orthoses and so on (14). 

Since using surgical and pharmacological treatments may be accompanied by a heavy economic burden and long-term consequences, finding effective non-surgical and non-pharmacological methods (such as exercise) is very helpful. Several studies have been conducted to investigate the effects of non-pharmacological treatments, especially exercise, as a way to treat or manage OA symptoms (15-17). 

It is worth noting that some studies have determined intense exercises to have an adverse role (18-21), although most of them agree that exercise with moderate intensity is ideal (17,22-24). Exercises of low and medium impact exert elastic and compressive forces on the joint cartilage. At this magnitude, tensile forces act as potent anti-inflammatory signals and inhibit interleukin (IL)-1β, tumor necrosis factor (TNF)-α and lipopolysaccharide-induced pro-inflammatory gene transcription as observed in studies including cartilage explants and *in vitro *systems (25-27). 

So far, studies on human models have had to evaluate the effects of treatment protocols using questionnaires or assessing some physical fitness measures. Hence, the effects of interventions on the joint’s cartilage remain ambiguous, because the researchers were unable to assess biochemical properties of the tissue *in vivo* (28). For this reason, there is a need to perform histopathological assessments that have to be done on animal models of OA resembling the condition in human models. According to our studies, intra-articular injection of monosodium iodoacetate (MIA) in animal models (such as rats), results in pathological changes closely resembling those seen in human OA (29). MIA injection into joints inhibits glyceraldehydes-3-phosphate dehydrogenase activity in chondrocytes, leading to disruption of glycolysis and eventual cell death (29, 30). So the aim of the present study was to examine the effects of moderate-intensity exercise on rats’ knee osteoarthritis.

## Materials and Methods

Eighteen male Wistar rats (173 ± 1 g, 8 weeks old) were obtained from the Pasteur Institute (Amol, Northern Iran). The maintenance and care of the experimental rats were in accordance with the guidelines of the Ethics Committee of Guilan University. Rats were kept in individual plastic cages in a 12:12 light-dark cycle (light-on period, 6:00 AM-6:00 PM) in a controlled temperature of 22 ± 2°C and 50±5% humidity on a sawdust bedding. They were fed a standard diet in pellet forms and had access to tap water *ad libitum*. Body weight was recorded at regular intervals. The animals were randomly divided into three groups (n = 6): Intact control, MIA only (OA), and training. 

The animals in training group were habituated on a motor-driven treadmill at a speed of 10m min^-1^ for 10 min/day for 1 week to reduce their stress regarding the new environment (17). After the adaptation period, a program of moderate physical training once a day, 5 days a week for 4 weeks with a speed of 18 m min^-1^ for 30 min/day had been performed (16). The training program started 24 hr after OA induction (17). For this purpose, the animals were anesthetized with ketamine (90 mg/kg, IP) and xylazine (20 mg/kg, IP); OA was induced by intra-articular injection of monosodium iodoacetate (Sigma-Aldrich) with a U-100 insulin needle containing 1 mg of iodoacetate diluted in 50 µl saline solution into animals’ right knee. In their left knee, 50 µl saline solution was injected (29).

On day 28, animals were killed by cervical dislocation under anesthesia. Whole knee joints were dissected, fixed in 10% formaldehyde solution in 50 cc vials and sent to a pathology laboratory. Histological procedures were done under the pathologist’s supervision. The samples decalcified with 5% formic acid, dehydrated through a descending series of ethanol with the use of an automated tissue processing apparatus. After embedding in paraffin, serial sections with a thickness of 7 µm were prepared for histological examination. Frontal and sagittal sections were prepared from tibiofemoral joints. The sections were stained with hematoxylin-eosin to observe the cellularity.

The severities of OA lesions were graded on a scale adopted from OARSI histopathology instructions. Three histopathological measures, including depth ratio of lesions (DR), total cartilage degeneration width (TDW) and significant cartilage degeneration width (SDW) were chosen for this purpose. DR is a measurement of the depth of cartilage degeneration (including areas of chondrocyte and proteoglycan loss, which may have good retention of collagenous matrix and no fibrillation) that is taken at the midpoint in each of the three zones across the tibial surface. TDW is the total width of the area of articular cartilage affected by any type of degenerative change (matrix fibrillation/loss, proteoglycan loss with or without chondrocyte death). And SDW is a measurement of the width of the tibial cartilage in which 50% or greater of the thickness (from surface to tidemark) is seriously compromised (31). Histomorphological scores in micrometers were assigned to these three measurements for statistical analysis (30).

The data were analyzed using the SPSS statistical software version 16. One-way ANOVA (*P*<0.05) and post-hoc Tukey test were used for the statistical analysis. 

## Results

***Depth ratio of lesions (DR)***

**Figure 1:**
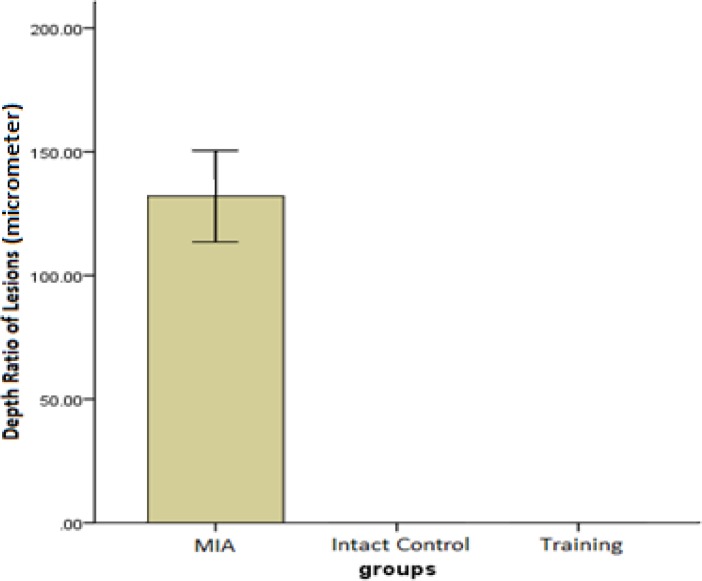
The mean ± SD of histological scores related to the depth ratio of lesions measured in micrometers. Higher scores show greater severity of lesions

For the measurements of DR, there were significant differences between the training and MIA group (*P*=0.001) (Figure 1).

***Total cartilage degeneration width (TDW)***

**Figure 2:**
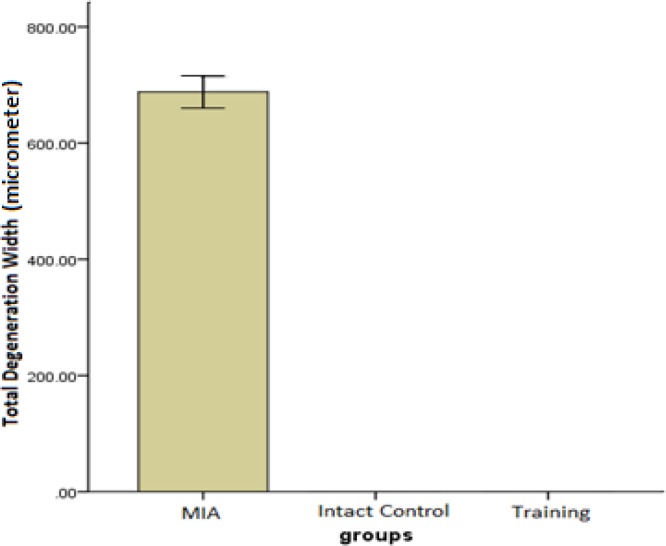
The mean ± SD of histological scores related to the total cartilage degeneration width measured in micrometers. Higher scores show greater severity of lesions

Significant differences were observed between the training and MIA group in the TDW measurements (*P*=0.001) (Figure 2).

**Figure 3:**
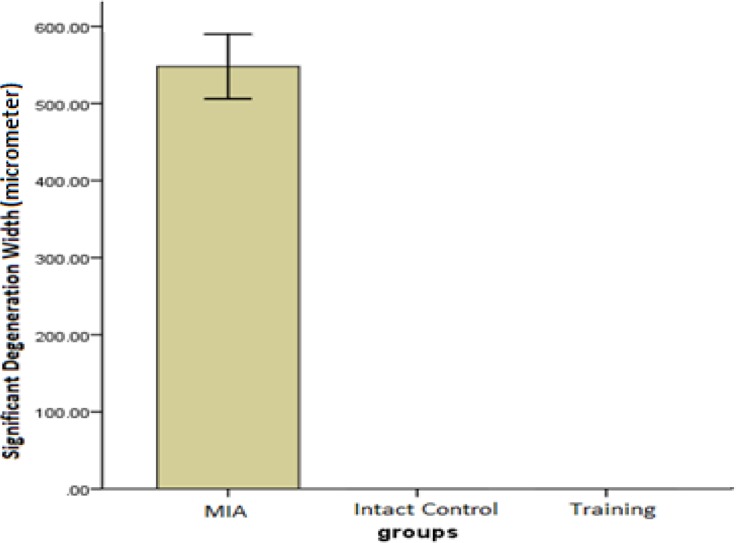
The mean ± SD of histological scores related to the significant cartilage degeneration width measured in micrometers. Higher scores show greater severity of lesions.

***Significant cartilage degeneration width (SDW)***

**Figure 4:**
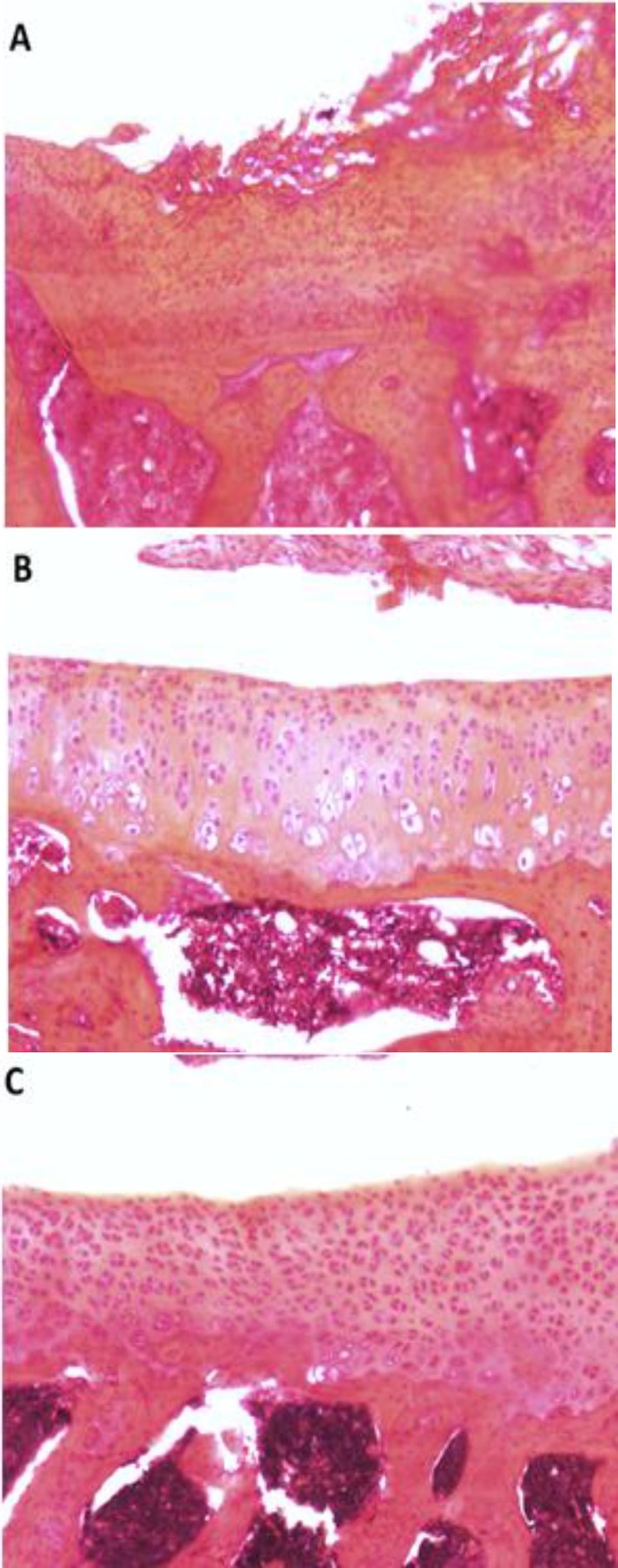
Photomicrographs of Histomorphological changes of knee joint sections of subjects in each experimental group. Cellularity and surface integrity was evaluated by Hematoxylin-Eosin staining (The original magnification was ×10). A: MIA only rat; OA lesions can be seen by tibial cartilage surface clefts and decrease in cellularity. Note the pink-red sites of lesions. B: Intact control rat; there are no observable changes in cartilage surface. C: Training rat; chondrocytes can be observed in many isogenic groups, which is the indicator of cell division stimulation

In terms of significant cartilage degeneration width, significant differences were seen between the training and MIA group (*P*=0.001) (Figure 3). Furthermore, photomicrographs of histomorphological changes of joint cartilage stained by Hematoxylin-Eosin for subjects in experimental groups are provided in Figure 4.

**Figure 5:**
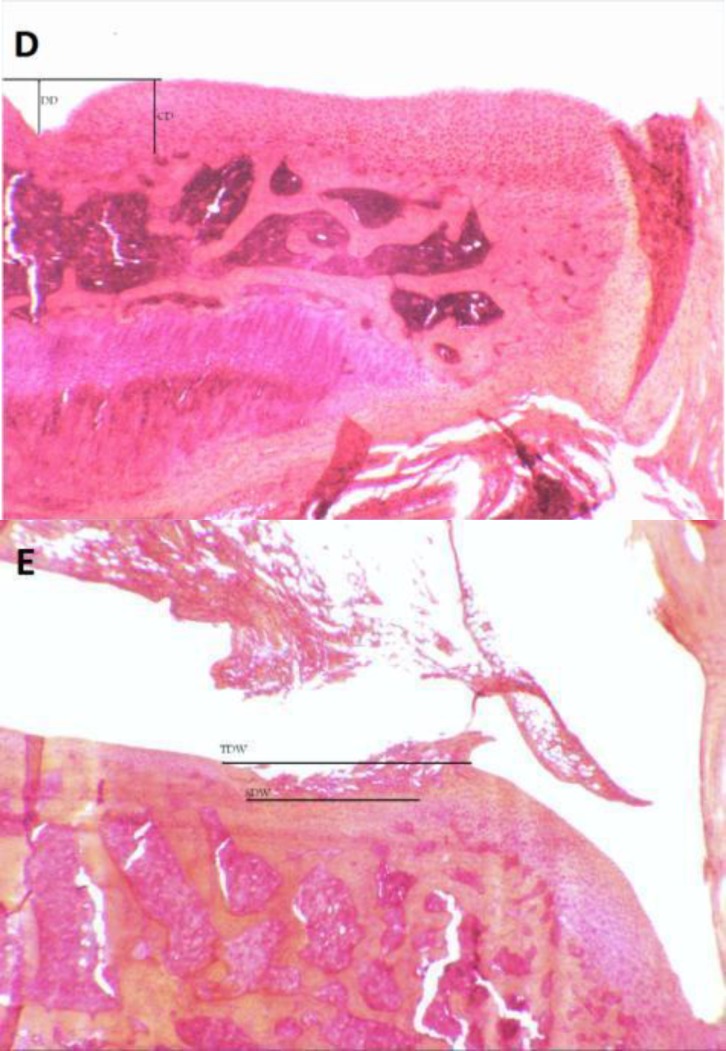
Method of histopathological assessment. D: Photomicrograph (The original magnification was ×3.2) showing a method for assessing depth ratio of Lesions; DD=Degeneration Depth, CD=Cartilage Depth, and DD/CD=Degeneration Depth Ratio; score 1 stands for the most severe lesion. E: Photomicrograph (The original magnification was ×3.2) showing a method for assessing Total Degeneration Width and Significant Degeneration Width; TDW= Total Degeneration Width and SDW= Significant Degeneration

Figure 5 provide methods for assessing histopathological measures used in the present study.

## Discussion

The present study is one of the few investigations that assess the effects of exercise on rats’ knee osteoarthritis. A four-week moderate intensity treadmill exercise was used as a treatment protocol. In order to induce osteoarthritic symptoms in rats’ knee joints, we used intra-articular injection of monosodium iodoacetate. This model is validated by other studies (32,33), but many caveats exist in utilizing the MIA models to represent human OA due to inherited variations, such as the biomechanical differences between two-legged and four-legged species (17).

The results of the present study show that a training protocol for 4 weeks could surprisingly almost treat OA symptoms of rats’ knee joint in 3 histological measures of depth ratio of lesions, total cartilage degeneration width, and significant cartilage degeneration width. In the training group, chondrocytes can be observed in many isogenic groups, which is the indicator of cell division stimulation (Figure. 4). Also, as presented in Figures 1, 2, and 3, training protocol used in the present study was beneficial in treating OA symptoms. From these Figures, it can be seen that the pathological score of the MIA group is nearly 130 micrometers for histological measure of depth ratio of lesions (Figure 1), 700 micrometers for total degeneration width (Figure 2), and 550 micrometers for significant degeneration width (Figure. 3). Whereas all three scores for the training group was zero and equal to healthy controls. 

The beneficial effects of this exercise program may be explained by its ability to suppress the signal transduction pathways of pro-inflammatory/catabolic mediators, while stimulating anabolic pathways. Mechanical strain of low magnitude inhibits inflammation by suppressing IL‐1β and TNF‐α‐induced transcription of multiple pro-inflammatory mediators involved in cartilage degradation. This also results in the up-regulation of proteoglycan and collagen synthesis that is drastically inhibited in inflamed joints (34).

These findings were also observed in a dose-response study by Galois *et al* (16), in which moderate exercise had positive effects on osteoarthritic joints of rats, even though intense exercise had converse effects. It seems that intensity, frequency and duration of aerobic exercise modulates chondrocyte response in some form, as seen in this study. The researchers suggested that the positive effects of moderate exercise could be related to a reduced level of chondrocyte apoptosis through anti-apoptotic capacities of stress-induced Hsp70 overexpression. 

Also another study that has been conducted by Cifuentes *et al* (17) indicated that a moderate exercise program could protect chondrocytes and increase defense mechanism against oxidative stress. They showed that this effect could be the result of increased activity levels of anti-oxidant enzymes such as superoxide dismutase (SOD) and myeloperoxidase (MPO).

The current treatments for osteoarthritis reduce pain and inflammation but have no significant effect on disease progression. In fact, effective treatments that would reverse the disease have not been developed (35), even though a limited number of studies have suggested that some anti-arthritic agents have the ability to arrest the pathologic process in humans and animals (36,37). Further research is required to establish the efficacy of such treatments. 

The mechanisms of OA are multifactorial and in order to treat this disease it is essential to find a way that not only can protects the cartilage against degenerative damage by stimulating its intrinsic repair capacity of chondroprotection, but also neutralizes the inflammatory/destructive potential of the mediators involved in oxidative-inflammatory stress as reactive oxygen species (ROS), nitric oxide (NO), proteolytic enzymes (such as metalloproteinases) and inflammatory cytokines (such as IL-1β) (38). The results of our study suggest that aerobic exercise with moderate intensity can successfully accomplish this goal.

 Cartilage, in fact, is an avascular tissue, and chondrocyte metabolism depends on diffusion and convection of synovial fluid for nutrition. Cyclic loading induced by physiological and overuse activities produces deformations, pressure gradients and fluid flows within the tissue. Mechanical stress has a direct effect on chondrocyte metabolism and can, under certain conditions, induce anti-apoptotic factors. Furthermore, compressive, tensile, and shear forces of appropriate/low physiological intensity also promote the up-regulation of proteoglycans and collagen synthesis, which are drastically inhibited in inflamed joints (38). Additionally, exercise promotes important changes in anti-oxidant enzyme activities, reducing oxidative damage and increasing tissue resistance against free radicals (39). Some studies have shown increases in SOD, Glutathione peroxidase and Catalase activities after aerobic exercise training in young rats (40, 41).

## Conclusion

Regarding the findings of the present study, it can be concluded that a moderate exercise program can significantly exert beneficial effects on osteoarthritic joints of rats. However, this claim has to be investigated further. One of the mechanisms for observing such effects may be the age of the subjects (8 weeks). Probably at this age, cartilage is more capable of repairing itself and thus, older rats were needed to induce more acceptable results. Therefore, we suggest that further studies be administered on examining the effects of this exercise protocol on older rats or by using other OA induction models (such as ACL or MCL transection and meniscectomy methods) and also with other exercise modalities such as aquatic exercises with different intensity, frequency and duration on both human and animal models. 
